# Developing a research database of primary aldosteronism: rationale and baseline characteristics

**DOI:** 10.1186/s12902-021-00794-7

**Published:** 2021-06-29

**Authors:** Wen Wang, Yuanmei Li, Qianrui Li, Tingting Zhang, Wei Wang, Dan Mo, Haoming Tian, Tao Chen, Yan Ren

**Affiliations:** 1 Chinese Evidence-based Medicine Center and CREAT Group, West China Hospital, Sichuan University, Chengdu, 610041 China; 2 Department of Endocrinology and Metabolism, Adrenal Center, West China Hospital, Sichuan University, Chengdu, 610041 China; 3 Health Management Center, West China Hospital, Sichuan University, Chengdu, 610041 China

**Keywords:** Primary aldosteronism, Hypertension, Research database

## Abstract

**Background:**

Management of primary aldosteronism (PA) has become a research hotspot in the field of endocrinology. To obtain reliable research evidence, it is necessary to establish a high-quality PA research database.

**Methods:**

The establishment of PA research database involved two steps. Firstly, patients with confirmation of PA diagnosis between 1 Jan 2009 to 31 Aug 2019 at West China Hospital were identified and data were extracted. Secondly, patients with confirmatory testing for PA will be enrolled into a prospective cohort. Data will be prospectively collected based on the case report forms since 1 Sep 2019. We evaluated the quality of research database through assessment of quality of key variables.

**Results:**

Totally, 862 patients diagnosed as PA were identified, of which 507 patients who had positive confirmatory testing for PA were included into the retrospective database. Among 862 patients diagnosed as PA, the mean systolic blood pressure (SBP) was 156.1 (21.7) mmHg, mean diastolic blood pressure (DBP) was 97.2 (14.5) mmHg. Among included patients, the mean serum potassium level was 2.85 (IQR, (2.47–3.36) mmol/L, and the mean plasma aldosterone concentration (PAC) was 28.1 (IQR, 20.0–40.4) ng/dL. The characteristics of patients with positive confirmatory testing for PA were similar. Validation of data extracting and linking showed the accuracy were 100%. Evaluation of missing data showed that the completeness of BMI (95.9%), SBP (99.4%) and DBP (99.4%) were high.

**Conclusion:**

Through integrating retrospective and prospective cohort of PA, a research database of PA with high quality and comprehensive data can be established. We anticipate that the research database will provide a high level of feasibility for management of PA in China.

## Introduction

Primary aldosteronism (PA) is a group of disorders which autonomous aldosterone secretion that is independent of renin, and cannot be suppressed by sodium loading [1]. As the leading cause of secondary hypertension, studies reported the prevalence of PA among patients with hypertension ranged from 5 to 20% [1–4], especial for patients with resistant hypertension, the prevalence were more than 20% [4].

Patients with PA may have poorer prognosis compared to patients with essential hypertension (EH). Studies suggested that PA may increase the risk of cardiovascular morbidity and mortality compared to patients with EH [1, 5–7]. A retrospective cohort study including 602 PA patients found that PA patients treated with mineralocorticoid receptor antagonists (MRAs) had approximately two-fold higher risk for cardiovascular outcomes, diabetes mellitus (DM), atrial fibrillation, and death [8]. A meta-analysis included 31 studies suggested that patients with PA had an increased risk of stroke, coronary artery disease (CAD), atrial fibrillation, and heart failure [6]. The current study demonstrated that even when patients with PA were treated with MRAs to achieve similar blood pressures as patients with EH with comparable cardiovascular risk profiles, they had an approximately two-fold higher risk for incident cardiovascular outcomes, atrial fibrillation, and death [8]. In addition, patients with PA have increased risk of metabolic disease including DM and obstructive sleep apnea-hypopnea syndrome (OSAHS) [8, 9].

Although modest progress has been made in case detection, diagnosis and treatment, the diagnosis and management of PA still has substantial challenges [1]. The methods for screening, confirmation and subtype classification of PA remain controversial [10]. The test of plasma aldosterone/renin ratio (ARR) is susceptible to drugs, diet, and serum potassium concentrations, and the optimal cutoff points for plasma aldosterone concentration (PAC) and ARR varied obviously among studies [11]. With respect to subtype classification, current diagnostic methods had limitation. The misdiagnosis of PA subtype based on CT/MRI was 37.8% [12]. As the recommend methods for subtype classification, adrenal vein sampling (AVS) requires considerable technical skill and has high rate of sampling failures. A study found that treatment of PA based on AVS did not show clinical benefits compared to treatment based on CT [5]. Moreover, current strategies for treatment of PA are still suboptimal. PA patients treated with MRA had higher risk for cardiovascular events, even achieving similar blood pressures as patients with EH [8]. The cure rate of hypertension among PA patients undertaking unilateral adrenalectomy were approximately 50% [13].

The optimal diagnostic methods and appropriate management of PA remain controversial. Lack of completeness and accuracy of data limited the production of high quality of evidence for patients with PA. Most published studies were retrospective studies and with small sample [11, 14, 15], with limited evidence to provide appropriate management strategies of PA, especial for Chinese population. Establishing a research database with complete and accurate information regarding PA is essential to provide sufficient evidence for PA. As one of the top hospitals in China, West China Hospital (WCH) has 4300 beds and covers patients from all over the country (http://english.cd120.com/outline/index.jhtml). There were about 260,000 discharged patients and nearly 200 patients diagnosed as PA in the year of 2018. Establishing a research database using electronic medical records (EMRs) system at WCH and prospectively collected information regarding long-term outcomes of patients with PA may provide valuable evidence on management of PA. Thus, in this study, we aimed to develop a research database for PA to provide better healthcare for PA in China.

## Methods

### Study overview

**Fig. 1:**
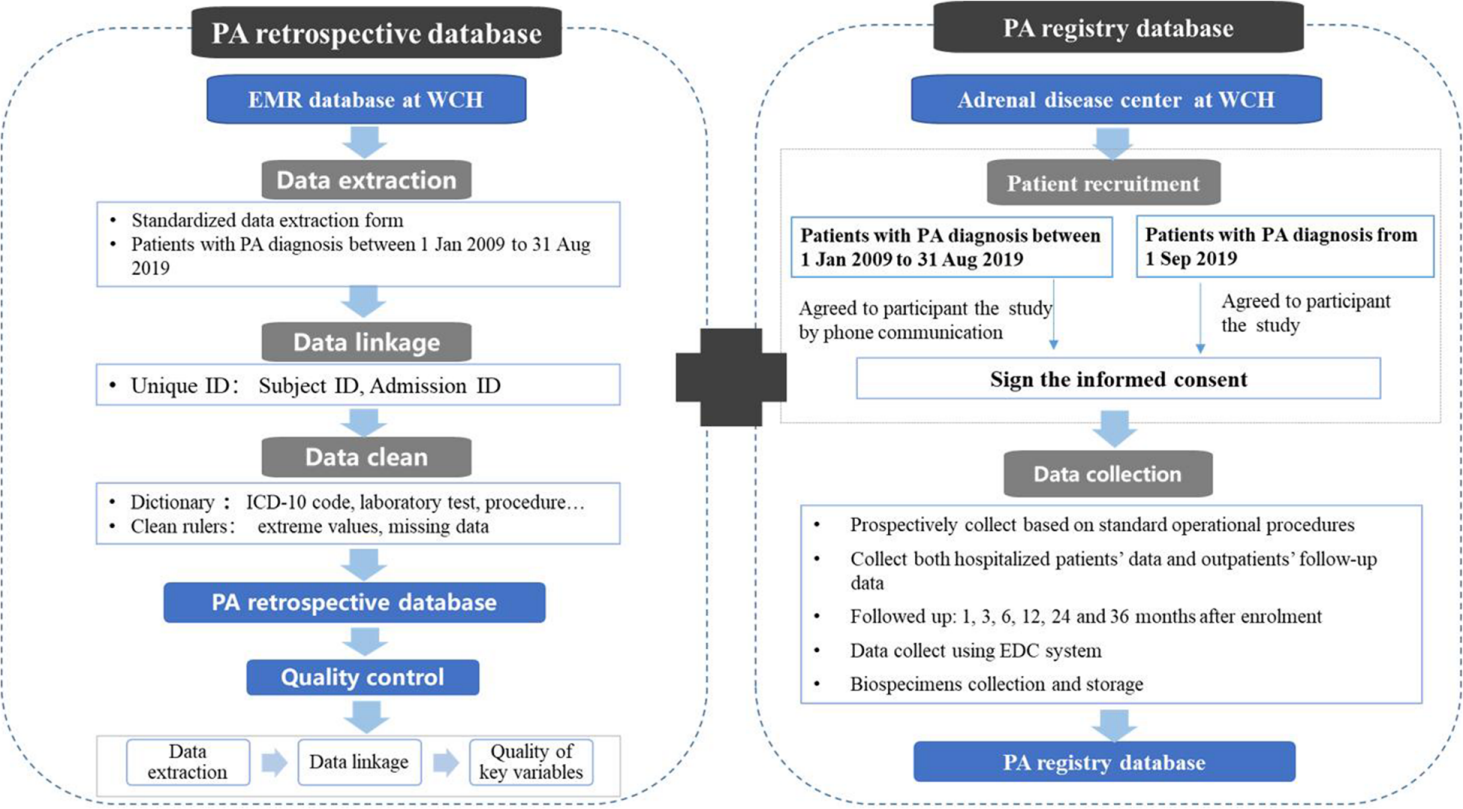
Development of PA research database in WCH

This study aimed to develop a research database for Chinese PA patients in WCH, to characterize disease progression and establish appropriate diagnostic criteria. The establishment of PA research database involved two steps. First, inpatients with diagnosis of PA and discharged from WCH between 1 Jan 2009 to 31 Aug 2019 were identified and information regarding clinical care were extracted from EMRs. Patients met the diagnosis criteria were finally included into the PA retrospective database. Second, we continuously recruit patients with confirmation of PA diagnosis at WCH from 1 Sep 2019 and data will be prospectively collected. For patients diagnosed as PA before 1 Sep 2019, a team of well-trained investigators contacted patients by either face to face or phone communication, and patients who agree to participant the prospectively cohort will be also recruited (Fig. 1).

The study was approved by the Ethical Committee of West China Hospital, Sichuan University (WCH2019–692). All patients recruited in the prospectively cohort will sign informed consent. With respect to PA retrospective database, data were exclusively obtained for routinely collected health data, which were collected for clinical purposes without specific a priori research goals. Therefore, the Ethical Committee of West China Hospital, Sichuan University approved to waive patient consent. All methods were performed in accordance with relevant guidelines and regulations (Declaration of Helsinki).

### Data sources

The PA research database was developed in WCH, Sichuan University. WCH is one of the largest single-site hospitals in the world, which located in Chengdu city, Sichuan Province (http://english.cd120.com/outline/index.jhtml). Initiated by the Department of Endocrinology and Metabolism, Adrenal Center of WCH was developed in the year of 2017. The center contained multidisciplinary departments including urology, nuclear medicine, laboratory medicine and radiology, and aims to improve the clinical care for patients with adrenal diseases. Up to now, 1500 patients with adrenal diseases were diagnosed and treated in WCH, of which nearly 1000 patients were with diagnosis of PA.

The EMRs system at WCH was initially developed in 2008, and has been proved a valuable data resource for clinical study [16–18]. The completeness of laboratory tests, such as plasma glucose and creatinine, were high [17]. According to internal reports in 2018, more than 99% records had international classification of diseases (ICD) coding, and validation of the ICD-10 code showed the accuracy were 88%.

### Study population

Patients who diagnosed as PA at WCH were included into the research database since 2009. Eligible patients should have PA related ICD codes or text terms such as “primary aldosteronism”, “aldosterone producing adenoma (APA)”, “bilateral adrenal hyperplasia”. In WCH, ARR was used for screening test, and patients with ARR > 20 were identified as possible cases of PA [19]. However, the performance of confirmation test and AVS changed over time. To addressed this important issue, we furtherly identified patients with at least one positive confirmatory test for PA including the captopril challenge test (CCT) and/or the saline infusion test (SIT). We defined positive confirmatory test according to the 2016 guideline of American Endocrine Society (TES) [1].

We excluded patients if they met either of the following criteria: (1) patients with incomplete information on date of birth, sex and discharged diagnosis; (2) without a valid patient identifier; (3) patients with other secondary hypertension such as Cushing’ syndrome, subclinical Cushing’ syndrome, congenital adrenal hyperplasia, renal arterial stenosis, multiple endocrine neoplasia (MEN), pheochromocytoma, and aldostrerone produing adrenalcorital carcinoma.

### Data collection and clean

The process of data collection was consisted with two steps (Fig. 1). Firstly, information experts extracted data from EMR systems based on the pre-designed, standardized data forms. Patients with diagnosis of PA were identified and information regarding clinical care of PA, including sociodemographic, diagnosis, laboratory tests, prescription and surgery were extracted. We linked data using unique identifiers including patient ID (a unique patient) and admission ID (a unique admission). Data clean was proceeded based on transparent and detailed data clean rules, which included variable dictionaries, standardize medical texts and approaches of missing data. Data clean rules were developed based on clinical rationales, experts’ advises and data distribution.

Secondly, patients who agree to participant a prospective PA cohort will be recruited and data will be prospectively collected based on standard operational procedures. Included patients will be followed up at months 1, 3, 6, 12, 24 and 36 after enrolment. Both hospitalized patients’ data and outpatients’ follow-up data will be collected. Sociodemographics, laboratory tests, imaging results will be extracted from EMR system based on pre-designed data forms, and information regarding symptom, signs and treatments will be recorded according to the case report forms (CRFs). Furthermore, we will additionally collect information regarding myocardial magnetic resonance, DICOM image of computed tomography adrenals and kidneys. Biological samples such as plasma will be also collected and stored at − 80 °C.

### Quality control

One hundred randomly selected medical chart were reviewed to assess whether the extracted and linked records were consistency with the original data. To ensure efficiency the accuracy of data collection, electronic data capture (EDC) system will be used for prospective data collection. Personnel training will be performed before the implementation of EDC system, and the access to the EDC system rights will then be granted. We also evaluated the proportion of missing data and outliers for key variables for the retrospective database.

## Results

Between 1 January 2009 and 30 August 2019, a total of 862 patients diagnosed as PA in WCH were identified and included into the retrospective database. Of these, 507 patients with at least one of the positive confirmatory tests.

### Characteristics of the study population

**Fig. 2:**
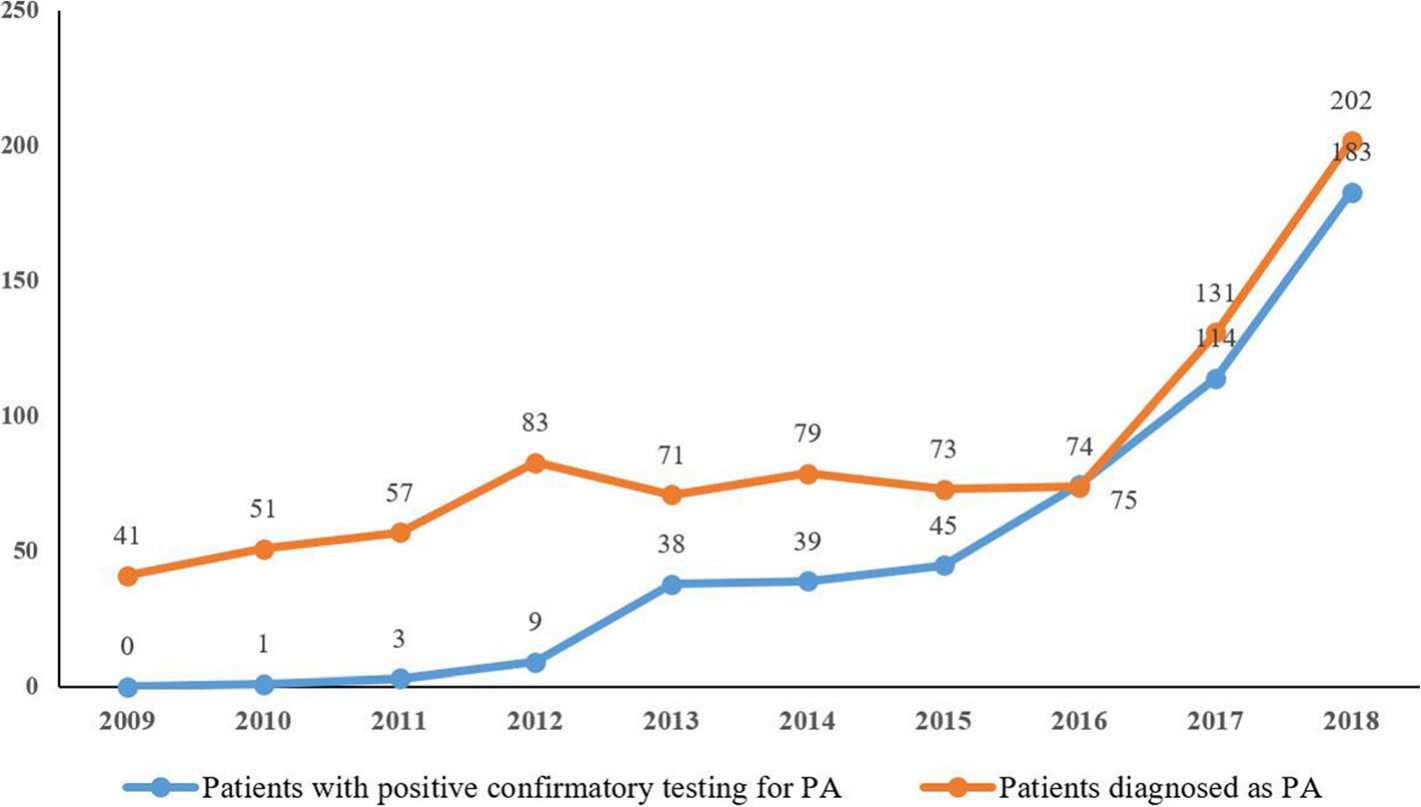
Admissions of the retrospective database over time

Of 862 patients diagnosed as PA, 648 (75.2%) were from Sichuan province, and 214 (24.8%) from other provinces. Diagnosis of PA was increased over time, especially after the establishment of the Adrenal Center of WCH in March, 2017. In the year of 2016, there were 75 patients with confirmation of PA diagnosis, but in 2018 the confirmed patients increased to 183 (Fig. 2).

Of these 862 patients with diagnosis of PA, 583 (67.6%) had positive ARR, and 507 (55.8%) had at least one positive confirmatory test of PA. Of 507 patients with positive confirmatory test of PA, 443 (87.4%) had positive SIT test, and 408 (80.5%) had positive CCT test. There were 479 (55.6%) patients with subtype classification, with 113 patients diagnosed by adrenal vein sampling (AVS) and 366 diagnosed by imaging. A total of 190 (22.0%) patients undertook AVS. Of these, 63 (33.2%) patients were considered as lateralization lesion, 50 (26.3%) were no lateralization lesion, and 77 (40.5%) with unsatisfactory or indeterminate AVS. There were 594 (68.9%) patients undertook adrenalectomy, and the operation rate was 73.2% before 2016, and 59.1% after 2016. Among 862 patients, 408 (47.3%) were diagnosed as aldosterone producing adenoma, and 72 (8.4%) diagnosed as bilateral aldosterone hyperplasia (Table 1).

**Table 1:** The diagnosis of patients included in to the retrospective database

	n/N (%)
**Patients diagnosed as PA**	862 (100%)
Positive ARR	583/862 (67.6%)
Screening not stated	279/862 (32.4%)
**Patients with confirmation test**	507/862 (55.82%)
Positive SIT	443/507 (87.4%)
Positive CCT	408/507 (80.5%)
Positive both test	344/507 (67.9%)
**Patients with alternative confirmation test**	355/862 (41.2%)
ARR > 20	76/355 (21.4%)
Confirmation not stated	279/355 (78.6%)
**Patients with subtype classification**	479/862 (55.6%)
**Adrenal vein sampling (AVS)**	190/862 (22.3%)
Unilateral disease	63/190 (33.2%)
Bilateral disease	50/190 (26.3%)
Unsatisfactory or indeterminate	77/190 (40.5%)
**Imaging (CT/MRI etc.)**	366/862 (%)
Unilateral adenoma	345/366 (94.3%)
Bilateral hyperplasia	21/366 (5.7%)
**Patients with a definitive diagnosis**	480/862 (55.7%)
**Aldosterone producing adenoma (APA)**	408/862 (47.3%)
By AVS + Patho	63 /408 (15.4%)
By imaging + Patho	345/408 (84.6%)
**Bilateral aldosterone hyperplasia**	72 /862 (8.4%)
By AVS	50/72 (69.4%)
By imaging	21/72 (29.2%)
By patho	1/72 (1.4%)

Among 862 included patients, 846 (98.1%) were Han nationality, 368 (42.7%) patients were male. There were 367 (42.6%) patients aged 18 to 45, 409 (47.2%) aged 45 to 60, and 85 (9.9%) aged 65 or older. The mean age was 47.8 (11.9) years old, and the mean BMI was 24.37 (8.4) kg/m^2^. Among 862 patients, 48 (5.5%) patients were diagnosed as chronic kidney disease (CKD), 21 (2.4%) as coronary artery disease (CAD), 118 (13.7%) as DM and 75 (8.7%) as OSAHS. The mean SBP among included patients was 156.1 (21.7) mmHg, of which 122 (14.2%) patients with SBP ≥180 mmHg. While the mean DBP was 97.2 (14.5) mmHg, and the proportion of patients with DBP ≥ 110 mmHg was 177 (20.5%). The median serum creatinine was 76.0 (IQR, 62.0–90.0) umol/L, and median A1C was 5.7 (IQR, 5.2–6.4)%. Among included patients, the median serum potassium concentration during admission was 2.85 (IQR, 2.47–3.36) mmol/L. The median supine ARR was 153.5 (IQR, 102.8–277.3), PRA was 0.49 (IQR, 0.22–1.04) ng/ml/h, and PAC was 28.1 (IQR, 20.0–40.4) ng/dL (Table 2).

**Table 2:** Baseline Characteristics of patients with PA discharged from the West China Hospital, 2009–2018

Characteristics	Patients with PA diagnosis ***N*** = 862	Patients with at least one positive confirmatory test of PA ***N*** = 507
**Age (yr), mean (SD)**	47.8 (11.9)	49.2 (11.8)
≤ 45 yr, n (%)	367 (42.6)	182 (35.9)
46-64 yr, n (%)	409 (47.2)	271 (53.5)
> =65 yr, n (%)	85 (9.9)	54 (10.7)
**Gender (male), n (%)**	368 (42.7)	213 (42.0)
**Race**		
Han	846 (98.1)	493 (97.2)
Other	16 (1.9)	14 (2.8)
**BMI (kg/m**^**2**^**), mean (SD)**	24.37 (8.4)	24.72 (3.7)
**Comorbidities, n (%)**		
CKD	48 (5.5)	37 (7.3)
CAD	21 (2.4)	13 (2.6)
DM	118 (13.7)	95 (18.7)
OSA	75 (8.7)	75 (14.8)
**Systolic Blood Pressure (mmHg)**	156.1 (21.7)	155.8 (20.9)
< 140	187 (21.7)	123 (24.3)
140–159	294 (34.1)	173 (34.1)
160–179	242 (28.1)	138 (27.2)
≥ 180	122 (14.2)	70 (13.8)
**Diastolic Blood Pressure (mmHg)**	97.2 (14.53)	96.2 (13.5)
< 90	266 (30.9)	166 (32.7)
90–99	209 (24.2)	144 (28.4)
100–109	194 (22.5)	103 (20.3)
≥ 110	177 (20.5)	91 (17.9)
**Serum Creatinine (umol/L)**	76.0 (62.0, 90.0)^a^	74.6 (61.0, 89.0)^a^
**HbA1C (%)**	5.7 (5.2, 6.4)^a^	5.7 (5.3, 6.5)^a^
**Serum Potassium (mmol/L)**	2.85 (2.47, 3.36)^a^	2.93 (2.53, 3.42)^a^
**Serum Aldosterone (ng/dL)**	28.1 (20.0, 40.4)^a^	26.4 (20.3, 36.5)^a^
**ARR (ng/dL)/(ng/ml·h)**	153.5 (102.8, 277.3)^a^	161.4 (64.3, 353.4)^a^
**PRA (ng/ml/h)**	0.49 (0.22, 1.04)^a^	0.42 (0.16, 0.88) ^a^

*CKD* chronic kidney disease, *CAD* coronary artery disease, *DM* diabetes mellitus, *OSA* obstructive sleep apnea-hypopnea syndrome, *BMI* body mass index, *ARR* Aldosterone-to-Renin Ratio, *PRA* plasma renin activity

^a^ reported as median (25th – 75th percentile IQR)

Among 507 patients with positive CCT and/or SIT test, the mean age was 49.2 (11.8) years old, and the mean BMI was 24.72 (3.7) kg/m2. There were 70 (13.8%) patients with SBP ≥180 mmHg, and 91 (17.9%) patients with DBP ≥ 110 mmHg. The median serum creatinine was 76.4 (IQR, 61.0–89.0) umol/L, supine ARR was 161.4 (IQR, 64.3–353.4), PRA was 0.42 (IQR, 0.16–0.88) ng/ml/h, and PAC was 26.4 (IQR, 20.3–36.5) ng/dL (Table 2).

### Quality of data

The successful linkage of HIS, LIS and PACS system using a unique identifier was 100%. Evaluation of missing data showed that the completeness of demography was high. The completeness of age, gender was 100%, and BMI among patients with positive confirmatory test of PA was 95.9%. There were less than 1% SBP and DBP data missing. With respect to laboratory tests, more than 99% of patients with positive confirmatory test of PA had at less one test of serum creatinine, serum potassium and LDL. The completeness in ARR and PRA were 85.8 and 84.0% respectively. Complements of key variables in PA retrospective database were summarized in Table 3.

**Table 3:** Complement of the key variables of patients with at least one positive confirmatory test of PA

Key Variable	No. patients with tests	Percent(%)
Age	507	100%
Gender	507	100%
BMI (kg/m2)	486	95.9%
Systolic Blood Pressure (mmHg)	504	99.4%
Diastolic Blood Pressure (mmHg)	504	99.4%
Serum Creatinine (umol/L)	506	99.8%
HbA1C (%)	232	45.8%
LDL (mmol/L)	506	99.8%
Serum Potassium (mmol/L)	507	100%
Serum Aldosterone (ng/dL)	507	100%
ARR (ng/dL)/(ng/ml·h)	435	85.8%
PRA (ng/ml/h)	426	84.0%

*LDL* low-density lipoprotein, *BMI* body mass index, *ARR* Aldosterone-to-Renin Ratio, *PRA* plasma renin activity

## Discussion

EMR system has received a lot of attention for its advantages on larger sample size and comprehensive individual-level health information regarding clinical care and outcomes. Recently, EMR has become a valuable data source for clinical studies worldwide [20, 21]. Through extracting data from EMR system at WCH, we have developed a PA retrospective database. The retrospective database included 507 patients with confirmatory diagnostic tests, with individual-level comprehensive information regarding clinical care such as diagnosis, laboratory tests, prescription and surgery. Validation of data governance suggested that consistency of data extracting and data linking with the original data was 100%. And the retrospective database showed high level of completeness and accuracy in key variables such as BMI, SBP and DBP. In addition, data will be prospectively collected using EDC system if patients agree to participant the prospective PA cohort. Through integrating PA retrospective database and prospectively collected database, a PA research database with high quality and comprehensive data will be established.

EMR has huge volume of data and detailed information regarding diagnosis, clinical care and outcomes, which is a valuable data resource for clinical care [21–23] . Integrate big data from EMR will be a powerful tool for investigating and improving health outcomes. In the field of PA, research database with large sample size and long period of follow-up have be developed, the database provide important evidence for management of PA [3, 8, 24, 25]. The German Conn’s Registry is a multicenter database aiming to evaluated the comorbidities and long-term outcome of patients with PA [24–26]. Patients with PA treated in Berlin, Bochum, Freiburg, Munich, and Wuerzburg were included since 1990. Several studies were conducted based on the Conn’s registry, and indicated that there were increased risk of comorbidities and cardiovascular mortality in patients with PA [24, 26]. The Japan Primary Aldosteronism Study (JPAS), a nationwide PA registry in Japan, included patients who diagnosed as PA and underwent AVS between January 2006 and October 2016 [27]. The registry has provided data to support a broad range of researches in PA in Japanese [7, 27, 28].

### Strengths and limitations

The research database has several strengths. Firstly, to the best of our knowledge, this is the first research database of PA in China. Secondly, the research database covers lots of patients diagnosed as PA. As a major healthcare system in West China, WCH provides tertiary care for the population resided in west China. The Adrenal Center of WCH is a unique center for PA in west China. Since 2009, there were nearly 1000 patients were with diagnosis of PA at WCH. Thirdly, the data quality of EMR are often suboptimal. The accuracy and completeness of data are the major concern. To address these concerns, we evaluated the completeness of variables in retrospective database, and the results suggested a higher level of completeness in important variables. In addition, most of registries for PA were retrospective, which may limit data quality [24, 27]. To ensure the accuracy of data and acquire long-term outcomes of patients with PA, we have also conducted a registry to prospectively collect based on standard operational procedures. Integrating retrospective database and registry, we expect to provide a useful data source supporting a wide range of PA epidemiology and public health research.

However, this study has some limitations. Firstly, although the database covers a relatively large number of patients with PA, it is a single-center database located in China, which limited the representative. Secondly, information were retrospectively collected between 1 Jan 2009 to 31 Aug 2019, which may affect the data quality. The diagnostic criteria for PA varied over time, and the diagnostic accuracy of PA was suboptimal before 2016. Nevertheless, we additionally identified a cohort of 507 patients with at least one positive confirmatory test of PA according to the 2016 guideline of American Endocrine Society. Thirdly, the laboratory examination method for Renin-Angiotensin-Aldosterone System (RAAS) may vary over time.

## Conclusion

In summary, the research database regarding PA integrating both retrospective and prospective cohort of PA may provide a valuable data resource for management of PA in China. We expect that the research database may bridge important knowledge gaps about PA both in China and around the world.

## Data Availability

Data will be available from the corresponding authors upon reasonable request.

## Abbreviations

PA: Primary aldosteronism
SBP: Systolic blood pressure
DBP: Diastolic blood pressure
PAC: Plasma aldosterone concentration
EH: Essential hypertension
MRAs: Mineralocorticoid receptor antagonists
DM: Diabetes mellitus
CAD: Coronary artery disease
OSAHS: Obstructive sleep apnea-hypopnea syndrome
ARR: Aldosterone/renin ratio
AVS: Adrenal vein sampling
WCH: West China Hospital
EMRs: Electronic medical records
HIS: Health information system
LIS: Laboratory information system
PACS: Picture archiving and communication system
ICD: International classification of diseases
APA: Aldosterone producing adenoma
CCT: Captopril challenge test
SIT: Saline infusion test
EDC: Electronic data capture
